# A radius valley between migrated steam worlds and evaporated rocky cores

**DOI:** 10.1038/s41550-023-02183-7

**Published:** 2024-02-09

**Authors:** Remo Burn, Christoph Mordasini, Lokesh Mishra, Jonas Haldemann, Julia Venturini, Alexandre Emsenhuber, Thomas Henning

**Affiliations:** 1 Max-Planck-Institut für Astronomie, Heidelberg, Germany; 2 Physikalisches Institut, Universität Bern, Bern, Switzerland; 3 Observatoire de Genève, Versoix, Switzerland; 4 Universitäts-Sternwarte München, Ludwig-Maximilians-Universität München, Munich, Germany; 5 Present Address: IBM Research, Rüschlikon, Switzerland; 6 Present Address: Physikalisches Institut, Universität Bern, Bern, Switzerland

**Keywords:** Exoplanets, Exoplanets, Exoplanets

## Abstract

The radius valley (or gap) in the observed distribution of exoplanet radii, which separates smaller super-Earths from larger sub-Neptunes, is a key feature that theoretical models must explain. Conventionally, it is interpreted as the result of the loss of primordial hydrogen and helium (H/He) envelopes atop rocky cores. However, planet formation models predict that water-rich planets migrate from cold regions outside the snowline towards the star. Assuming water to be in the form of solid ice in their interior, many of these planets would be located in the radius gap contradicting observations. Here we use an advanced coupled formation and evolution model that describes the planets’ growth and evolution starting from solid, moon-sized bodies in the protoplanetary disk to mature Gyr-old planetary systems. Employing new equations of state and interior structure models to treat water as vapour mixed with H/He, we naturally reproduce the valley at the observed location. The model results demonstrate that the observed radius valley can be interpreted as the separation of less massive, rocky super-Earths formed in situ from more massive, ex situ, water-rich sub-Neptunes. Furthermore, the occurrence drop at larger radii, the so-called radius cliff, is matched by planets with water-dominated envelopes. Our statistical approach shows that the synthetic distribution of radii quantitatively agrees with observations for close-in planets, but only if low-mass planets initially containing H/He lose their atmosphere due to photoevaporation, which populates the super-Earth peak with evaporated rocky cores. Therefore, we provide a hybrid theoretical explanation of the radius gap and cliff caused by both planet formation (orbital migration) as well as evolution (atmospheric escape).

## Abstract (web summary)

A prominent under-density in the observed radius distribution separates small exoplanets in two categories. The study demonstrates, through planet formation and evolution simulations, that the larger planets, whose composition has been disputed, may be water-rich planets migrating towards the star, where they become steam worlds.

## Main

The driving process leading to the underabundance of planets with radii *R* ∼1.7 *R*_⊕_ found in the data from the Kepler spacecraft^1–3^ could be related to escape, due to stellar X-ray and ultraviolet irradiation^4–6^ or core-powered mass loss^7–9^, of hydrogen and helium (H/He) on top of a core consisting of silicates and iron. In the following, we call this mixture of solid materials rocky. With this limited compositional inventory, the observed sharp drop-off of the sub-Neptune occurrence at radii greater than ∼3 *R*_⊕_—the radius cliff—likely requires an additional mechanism, such as H_2_ sequestration in the magma ocean^10^.

Another possible explanation for the radius distribution is that the sub-Neptunes are water-rich, with water mass fractions of several tens of percent^11–13^. Such large water contents are a consistent prediction of planet formation models that include the effect of planet–disk interactions, leading to migration of ice-rich planets from outside the water condensation line towards the star^14,15^. In this scenario, the common assumption that the gaseous envelopes are dominated by H/He is no longer required. This hypothesis has recently gained supporting observational evidence based on planets around M stars^16^, whose bulk density distribution can be well reproduced with silicates and iron for super-Earths and about equal fractions of rock and water for sub-Neptunes. The tentative evidence for this scenario lies in the small scatter of sub-Neptune densities^16^, which might not be expected for rocky cores with a H/He envelope. However, this can also be matched in the classical picture if the initial H/He fraction of the planets exhibits little scatter at fixed planetary mass due to formation and boil-off processes^17^.

For more massive stars, the smaller observational signal often impedes precise planetary mass determination. In that case, a clear picture of density does not emerge with present-day data, and the different theoretical models cannot easily be falsified^18^ unless the planetary masses are constrained using theoretical arguments, such as the output of a planet formation model^15,19,20^.

In the case of water-dominated outer layers of the planet, the phase of water determines the precise radius. For close-in planets, water forms a hot—to a large degree supercritical—vaporized hydrosphere, here called the steam envelope^12^, which increases the radius compared to condensed, solid, high-pressure ice^21^. Due to model limitations, water was not consistently included in this lower-density phase in the first works coupling a planetary mass distribution from planet formation to photoevaporation models^5,19^ and/or comparing the observed valley locus with the theoretically predicted one^19,22^.

This prior work excluded that the super-Earths contain substantial amounts of water and favoured water-poor sub-Neptune compositions because planets containing solid ice were located in the radius gap. Later, a combined formation and evolution model with the correct water phases demonstrated the emergence of the radius valley as a separator between dry and wet planets formed within, respectively beyond, the ice line^15^. In that work, the main driver of the dichotomy is varying pebble accretion efficiency based on pebble composition, which produces smaller rocky and larger icy cores. Recently, another study^20^ reached the same conclusion using a similar model, incorporating also *N*-body interactions between planets.

Here, we use a coupled formation and evolution model to synthesize a population of planets that can be statistically compared to the Kepler mission by applying its detection bias^23^. During the evolution stage, the planets are evolved individually by calculating their interior structure, which captures effects of cooling and contraction. In addition, for the nominal model, we assume that water mixes with H/He at the high temperatures above the runaway greenhouse limit relevant for comparison to the Kepler data^24,25^. For water, we use a new equation of state^26^, which covers all possible phases. Under these assumptions, water is also present in the upper layers of the gaseous envelope or can even be the only volatile constituent. Thus, we weight by mass the loss of H/He (following ref. ^27^) and water^28^ while keeping the envelope water fraction (or metallicity) constant—an approximation most accurate in the limit of efficient hydrodynamic mass loss^29^.

The formation stage is taken from our global planet formation modelling^30,31^ growing 1,000 systems of planets. They were generated in a statistically robust way by choosing initial conditions randomly sampled from observations of disks^32^. This enables us to extract the key mechanisms shaping the radius valley and statistically quantify their ability to reproduce observations. In particular, orbital migration and *N*-body interactions between growing planets are included. Motivated by prior *N*-body simulations^33^, which show that some late dynamical events can influence the inner planetary systems, we extended the formation phase with *N*-body integration and planetesimal accretion to 100 Myr, compared to 20 Myr in the original work^31^. We note that planetesimal accretion essentially terminates after the gaseous disk has dissipated at around 3 Myr due to the missing gas drag damping the planetesimals’ eccentricities and inclinations. The formation and evolution processes, detection bias and their implementation are detailed in the Methods section.

## Results

### Radius distribution

**Fig. 1:**
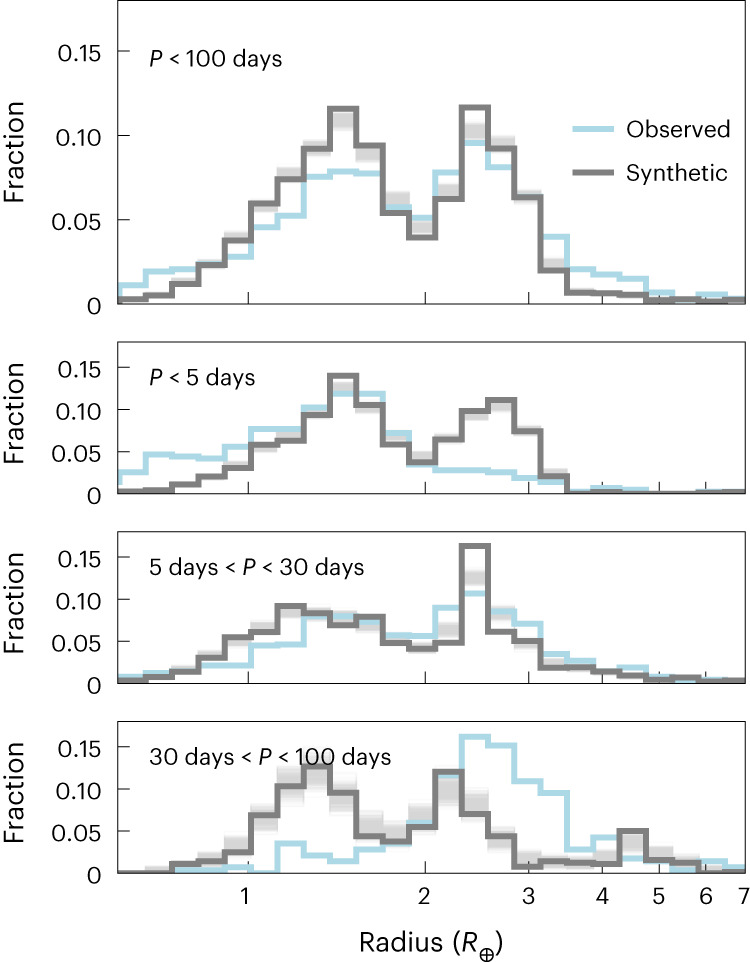
Radius histograms of observed and synthetic planets. The light blue line shows the observed distribution without correction for the observational bias^2^ and the grey line the synthetic one using the updated, nominal evolution model with the bias of the Kepler survey applied. Opaque lines show 500 random realizations of the synthetic planets with 5% error in radii. We restrict the sample to planets with certain orbital periods (*P*), as indicated in the top-left corner of each panel. Histogram bin counts are normalized by the total number of planets in the different samples.

**Extended Data Fig. 1:**
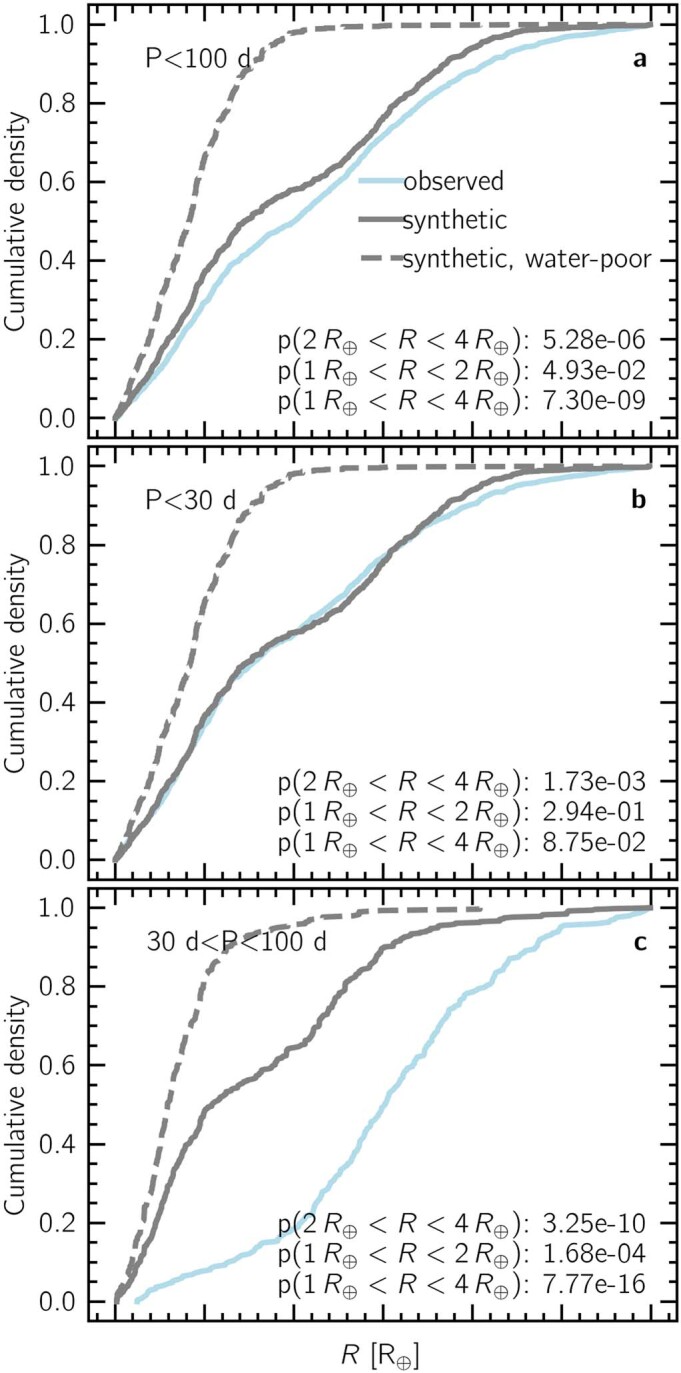
Cumulative radius distributions of observed and nominal, synthetic planets (R < 4 R_⊕_) in different orbital regions. **a**–**c**, Distributions at different orbital period ranges marked in the top left. Two-sample, Kolmogorov-Smirnov (KS) test p-values are listed in the bottom right. The null hypothesis that the observed and the synthetic distributions are the same can be rejected for the region outside of 30 day orbital periods and for all sub-Neptunes but not for close-in super-Earths (p-values above 0.05). This agreement leads to a p-value above 0.05 for the whole radius range if only planets on short orbits are considered. In clear disagreement with observations is the synthetic sub-sample of planets with less than 50% water mass content in the envelope (dashed).

We contrast our theoretical radius distribution with applied observational bias to observations^2^ in Fig. 1. For the overall distribution, we find an excellent match for the locus of the sub-Neptune peak, the radius valley and the super-Earth peak. The synthetic radius cliff is only marginally steeper, while the relative number of super-Earths in the model is higher than observed. Statistical quantification of the differences (Extended Data Fig. 1) reveals that the radius distribution of super-Earths within 30 days is well matched. At larger distances, we synthesize and theoretically ‘detect’ more rocky planets than observed. The depth of the radius valley is marginally deeper in the synthetic population even after accounting for typical measurement uncertainties^3^. At the innermost and larger orbital periods, differences between theoretical and observed distributions offer insights for model improvement, as discussed in the following sections. We achieved a natural match with the observed distribution of planetary radii by consistently including more realistic physics, especially various phases of water, compared to previous works^19^. The result is obtained from following the self-consistent formation and evolution of initially 100 0.01 *M*_⊕_ planets per disk. We did not adjust any parameters in the underlying formation phase or the evolutionary model.

**Fig. 2:**
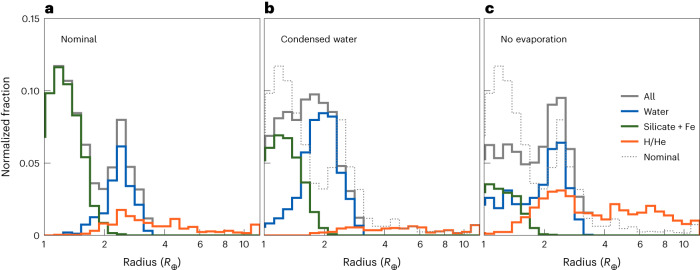
Radius histograms of synthetic planets with orbital periods shorter than 100 days for different model assumptions without any bias applied. **a**–**c**, Full distribution is shown in grey, and coloured histograms show different compositional subsets (pure rocky in green, water-rich without any H/He in blue, with some H/He in orange): nominal model (**a**), water assumed to be condensed out in a solid ice layer resistant to evaporation below H/He (**b**), nominal treatment of water but excluding evaporation (**c**). The dotted histograms show the full distribution from the nominal simulations. As in Fig. 1, bin counts are normalized by the total number of planets in the different samples.

Figure 2 displays radius distributions of different modelling runs without observational bias applied, categorized by bulk composition. Figure 2a shows that the valley separates smaller, dry planets from lower-density, vapour-enveloped, wet planets with little overlap. The planets whose envelopes contain some H/He make up only 13–16% (depending weakly on age) of the sub-Neptunes. If water-rich planets are excluded, we do not reproduce the observed radius distribution (see also Extended Data Fig. 1). Furthermore, most of the planetary envelopes of sub-Neptunes with some H/He (orange), contain less than 10% of H/He by mass.

The radius cliff located at ∼3 *R*_⊕_ is in our simulations a second compositional transition from planets containing ≳90% water to those that contain several tens of percent of H/He in mass. It is accompanied by a corresponding drop in the occurrence of planets at ∼20 *M*_⊕_. Thus it is a feature that emerges due to enhanced gas accretion onto more massive planets.

We further varied model assumptions to understand how robust the results are. First, we found them to be insensitive to any bloating mechanism (Methods). Second, under the assumption that water is buried below the H/He envelope and is forced to remain in condensed form, we do not reproduce the radius valley (Fig. 2b). Instead, water-rich cores populate the radius range where the observed valley is located. This is in agreement with previous works^19,22^ and shows that the cause of recovering a radius valley with water-rich planets can be attributed to the (correct) phase of water and its distribution within the envelope.

As a third variation, Fig. 2c shows the case excluding atmospheric mass loss while keeping the water mixed into the H/He envelope. There, we get a distribution that is not in agreement with the presence of a radius valley. Instead, we obtain many (low-mass) large planets with a rocky core and a H/He envelope and also a distribution of water-rich planets that smoothly extend to low radii. In reality, both of these kinds of planets would be unable to retain most of their volatile envelopes. For the same reason, too few rocky planets exist. This highlights the need for atmospheric escape shaping the distribution of planetary radii even for water-rich compositions. It is necessary to populate the super-Earth peak with rocky planets by stripping their H/He envelopes. We conclude that the valley is a hybrid consequence of both formation (migration leading to the sub-Neptune peak) and evolution (evaporation leading to the super-Earth peak).

### Dependency on orbital period

**Fig. 3:**
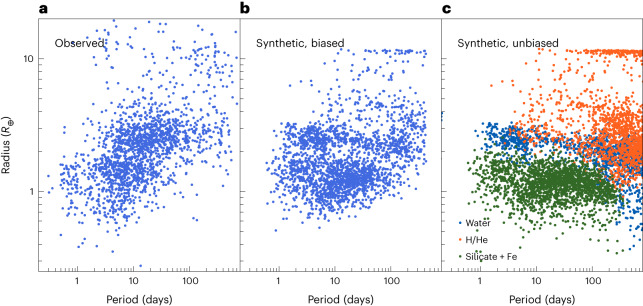
Transit radii as a function of orbital period. **a**, Observational data from the California Kepler Survey^2^. **b**, Synthetic data with applied observational bias using KOBE (Methods). **c**, Unbiased synthetic distribution colour-coded by composition (as in Fig. 2).

In addition to radii, orbital periods of exoplanets can be determined precisely. Figure 3 shows the period–radius distribution of observed^2^ (Fig. 3a) and modelled (Fig. 3b,c) exoplanets. Qualitatively, the observed distribution matches the synthetic distribution with applied observational bias. We apply the bias of the full Kepler survey without taking into account that not all planets are included in the California Kepler Survey catalogue^2^. Therefore, more numerous points appear in the middle panel. In both the observed and the synthetic distribution, the radius valley can be made out at comparable locations.

Figure 3c shows the synthetic data without observational bias applied. Additionally, the composition is colour-coded. Rocky Earths and super-Earths (green) are found at small radii below the valley. This distribution is truncated at 300 days, where the equilibrium temperature is close to 300 K.

The planets with water but without H/He (blue) fill the parameter space at radii greater than the rocky planets above the valley and at larger distances. Planets with H/He (orange) populate the even larger radii and are also more common at lower radii further from the star.

**Extended Data Fig. 2:**
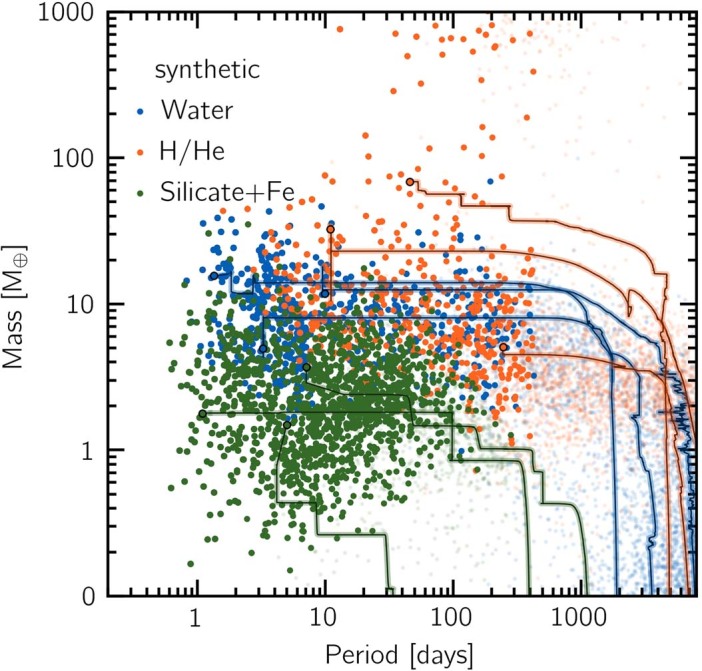
Orbital period against masses of synthetic planets after 5 Gyr of evolution. Planets observable and non-observable with the Kepler space telescope are shown (KOBE bias applied) as opaque or transparent points respectively. The points are colored as in Fig. 3 and we show in addition the formation tracks of a selection of planets from the different categories as lines. Sudden jumps in mass are caused by giant impacts. Since there is no effect of the lower density material on the planetary mass, the radius valley is not visible in mass space.

As revealed by the selected formation tracks in Extended Data Fig. 2, this pattern is shaped by migration and collisions during the formation stage as well as photoevaporation. Rocky planets generally form at short distances inside the water snowline. They grow first by planetesimal accretion and then (and most importantly) by giant impacts with other rocky protoplanets. However, their growth is limited by the number of building blocks inside the ice line. Due to their small mass, little orbital migration occurs inside the ice line.

At larger distances to the star, more solids are available for accretion, given the assumed radial power-law slope of −1.5 for the planetesimal disk surface density. Also, the condensed icy material outside the water–ice line can be accreted as solid. This leads to promoted planetary growth up to several Earth masses, where migration is more efficient^34^. Thus, planets migrate to the inner region, where they collide with smaller rocky planets, especially when the gas disk dissipates and gas-induced eccentricity damping ceases. Collisions with other bodies—or, at the closest distances, the radiation flux of the host star—can lead to the expansion and Roche lobe overflow of the gaseous envelope and thus remove the H/He content. However, due to limitations in our model, this is not the case for the heavier water. Only after switching to the evolution stage do we assume that the constituents mix perfectly. These processes therefore give rise to a population of planets with pure water envelopes. More massive planets or those located at larger distances share a similar formation history but are not stripped completely of H/He (orange planets and tracks in Fig. 3 and Extended Data Fig. 2).

During the evolution stage, planets cool and are subject to photoevaporation, which reduces their size. The most frequent evolutionary pathway is the loss of a (pure) H/He atmosphere from a rocky core. A mixed or water-dominated envelope can also be lost completely, resulting in a bare, rocky core. This happened for 17% of the (eventually) rocky super-Earths in the biased, synthetic population, which had at least 10% water by mass after the formation stage. This outcome occurs for the lightest migrating planets.

**Extended Data Fig. 3:**
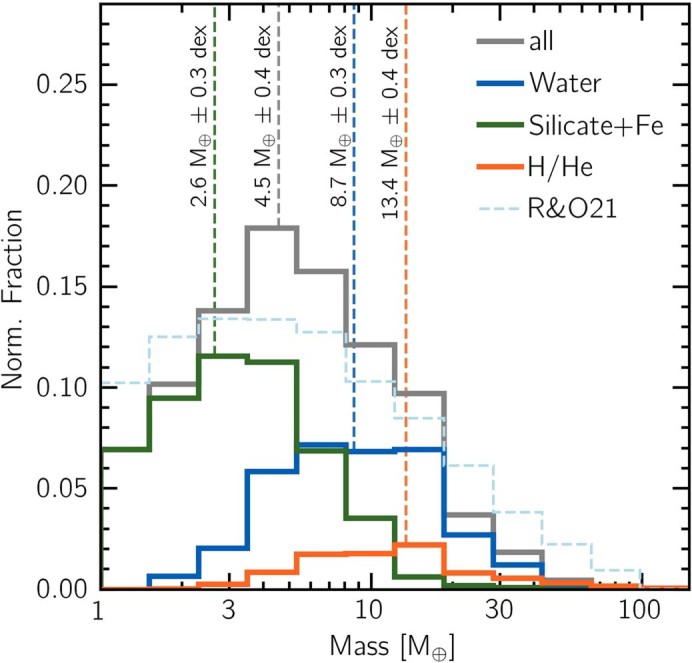
Mass histogram of the nominal simulation with applied Kepler bias. Lines show different groups: all planets (gray), rocky planets (only iron core and silicate mantle, green), planets with water but no H/He (blue), and planets with H/He (orange). Dashed lines mark logarithmic mean values of each category and are labeled with mean and standard deviations. The distribution of core masses retrieved by Rogers and Owen^18^ (R&O21) in a pure photoevaporation scenario is shown for comparison.

Although there is an overlap in mass between volatile-rich and rocky planets (Extended Data Fig. 3), the overall formation pathway occurs along the following lines: rocky cores are lower-mass planets that formed almost in situ by a giant impact stage, while volatile-rich planets are more massive and for that reason migrated substantially to their present-day location. Low-mass, volatile-rich planets at large distances, in contrast, do not migrate towards the host star (which would fill the valley), because type I migration is slower for lower planet mass^34^.

## Discussion

### Mass distribution and mass–radius diagram

A fundamental property of planets with regards to their formation is their mass. We obtained the mass distribution, shown in Extended Data Fig. 3, by modelling planet growth from the embryo stage onward via solid and gas accretion followed by long-term evolution. This forward modelling approach differs from (and allows for contrasting insights compared to) using an educated guess for the planetary masses^35^ or retrieving mass distributions by solving the inverse problem and starting with radii found by the Kepler satellite^18,35^. We obtain a trend in mass from low-mass, rocky planets over more massive, migrated, steamy water worlds to the H/He-rich (sub)giants. However, the mass distributions of each kind of planet are broad and overlap, which results in a unimodal total mass distribution.

The characteristic masses are a few Earth masses for rocky planets and about ten Earth masses for water worlds. These mass scales can be understood analytically by recalling that most of the rocky planets went through a giant-impact phase after a destabilization of the systems. The appropriate mass scale then depends on the number of solid building blocks in a dynamically enhanced feeding zone during the giant-impact phase^36^. In contrast, for the migrated, water-rich sub-Neptunes, the equality of migration against growth or, if present, the saturation of corotation torques sets the mass scale^36^.

This implies that the mass of the rocky planets depends on the solid accretion mechanism, while the mass of migrated steam worlds is less sensitive to it. Independently from our work, a study^15^ using a global model with pebble accretion instead of planetesimal accretion resulted in a mass distribution with more distinct separation of rocky and water-rich planets—that is, a bimodal distribution—in mass. An overall mass distribution similar to ours was retrieved in a Bayesian framework^18^ by fitting the radius distribution of Kepler planets, as shown in Extended Data Fig. 3 (dashed). This favours the unimodal distribution, although a low probability of planets containing water was inferred by the authors. We attribute it to their assumption that water is in the condensed phase at all temperatures.

**Extended Data Fig. 4:**
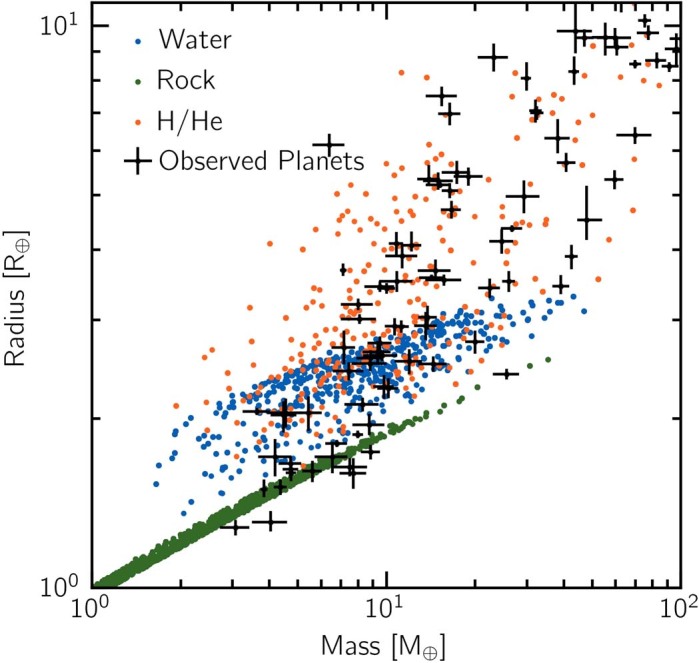
Total planetary masses against planetary radii of observed and synthetic planets. The observational data was taken from the NASA Exoplanet Archive (composite data, accessed on Dec 9, 2022) filtering for relative mass, stellar mass, and radius standard errors smaller than 20% and stellar masses ranging from 0.75 to 1.25 *M*_⊙_. Errorbars show the standard error. We caution that this sample of planets does not come from a homogeneous survey.

In Extended Data Fig. 4, we also show the synthetic mass–radius diagram for the nominal model. We find that our model leads to planets covering the area occupied by precisely characterized, observed planets around Solar-type stars. A tentative overdensity of both observed and simulated planets is located close to the lowest-density planets without H/He (uppermost blue points in the diagram). This would be in agreement with recent observational findings^16^ for M stars but is for Solar-type stars without statistical significance. So far, no unbiased statistical sample of characterized planets with precise mass and radius measurements exists for a more detailed comparison.

### Potential reasons for discrepancy of close-in, wet planets

While the synthetic and observed radii match well, we find a difference in the orbital period distribution of the close-in sub-Neptunes. In Fig. 3, by comparing the observed (Fig. 3a) with the synthetic, biased (Fig. 3b) population, it becomes apparent that a group of sub-Neptunes is theoretically predicted at orbital periods of 1–5 days but is absent in the observed sample. Instead, a similar number of planets are missing at longer orbital periods. It is expected^37^ that even at high irradiation, steam envelopes can be kept for sufficient core masses. However, with the exception of 55 Cancri e^38^, there are no observed planets with low bulk densities in this regime. Thus, the very close-in sub-Neptune population that we obtain is disfavoured by observations.

A possible explanation is that their migration might halt at greater orbital distances than the model predicts. Currently, the inner edge of the gas disks, placed at corotation radii of observed young T Tauri stars^39^, acts as migration trap. Since sub-Neptunes form early in the gaseous disks (to be able to migrate substantially), and given a sufficient source of disk turbulence in the inner disk, viscous heating would be efficient. Therefore, the location of thermal ionization—that is, the inner edge of the dead zone to the magnetorotational instability^40^—can lie further from the star. This introduces a migration trap^41,42^ and can cause the distribution of migrated planets to peak at larger orbits. Future formation models should include the physical transition in the strength of magnetorotational turbulence together with tracking the heavy-element content in the gaseous phase. In addition to a potential resolution to the discrepancies, this will enable a more detailed discussion of the radius valley’s slope and time dependency in the steam-world scenario for sub-Neptunes.

**Fig. 4:**
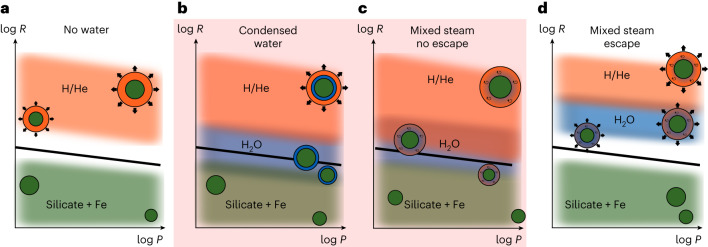
Schematic visualization of the four considered scenarios. The location of the observed radius valley is represented as black line, and the planetary population of pure silicate and iron bodies (green), water-rich planets (blue) and H/He gas giants (orange) are shown as blurred rectangles. Example interior structure schematics are shown with indicators for mixing and escape. **a**,**d**, We identified in this work that the radius valley is shaped by either a mass-loss mechanism without any water on the planets (**a**) or photoevaporation combined with water in supercritical steam envelopes (**d**), which is in better agreement with the predictions of formation theory, in particular that planets should be undergoing orbital migration. **b**,**c**, Neither the scenario with condensed water (**b**) nor that without photoevaporation (**c**) reproduces the observed valley, and are thus marked in red.

To summarize, by coupling a global, end-to-end planet formation model with orbital migration and *N*-body interactions to different evolution pathways, we identify two scenarios (Fig. 4a,d) that lead to a distribution of planetary radii consistent with the observed location of the radius valley^1^. A key aspect is the state of water, which in this work is in the correct phase—that is, typically supercritical vapour—which we assume to be mixed with H/He.

In this way, we provide theoretical support for the scenario of formation or, more specifically, gas-driven orbital migration, shaping the distribution of mostly water-rich, steam-envelope planets populating the sub-Neptune peak (Fig. 4d). Only at larger planetary radii do H/He become the dominant gaseous constituents. This is in agreement with an earlier study^15^, albeit the formation mechanism assumed in that study was pebble accretion. The bimodal mass distribution (below 20 *M*_⊕_) shaped by pebble accretion and its isolation mass is not required to match observed radii. Instead, a unimodal mass distribution can reproduce the observations.

Assuming our model’s mass distribution is approximately correct, we can further falsify a condensed-out ice layer scenario (Fig. 4b). In any case, at the observed planets’ equilibrium temperatures, water is not in the solid ice form^21,25^.

At the same time, we also give evidence for the necessity of an evolutionary mass-loss mechanism. Atmospheric escape is necessary to populate the super-Earth peak with evaporated rocky cores. Without evaporation, rocky planets with small H/He atmospheres that form during the gas disk stage inside the ice line would lead to low-mass planets with large radii, resulting in a radius distribution inconsistent with observations (Fig. 4c).

Our results are not distinctly sensitive to modifying the photoevaporation model or the presence of a bloating mechanism. With both orbital migration and atmospheric escape causing the radius valley, one can speak of a hybrid origin of the radius valley caused by both formation and as evolution. However, we were limited by the availability of detailed and fast models of photoevaporation of high-metallicity envelopes to further explore the effects of chemical fractionation due to mass loss or molecular cooling, which need to be developed in the future to study the sub-Neptune population in further detail^29,43^.

Finally, based on pure planet evolution calculations, we cannot rule out the classical picture of photoevaporation of H/He envelopes on top of exclusively rocky cores^6^ as the only mechanism shaping the radius distribution. However, many different planet formation models consistently predict the migration of ∼10 *M*_⊕_ water-rich planets to regions close to the host star, a prediction already made, for example, in the first generation of population syntheses^14^. We show here that the resulting population of close-in, water-rich planets can agree with the observed valley. If the prediction of the presence of water-rich sub-Neptunes should turn out to be incorrect, it would call for a revision of fundamental aspects of formation theory. These aspects could, for example, be protoplanetary disk structures leading to less orbital migration^44,45^ or a high efficiency of volatile loss during planet assembly^46^.

Today, observational constraints on the bulk composition of sub-Neptunes are still inconclusive^47–49^, but with the help of the James Webb Space Telescope and the future ARIEL mission, we expect to find more evidence for either the water-rich or the H/He-dominated composition and advocate the investigation of sub-Neptune atmospheres to resolve the mysteries of the radius valley.

## Methods

### Formation model

The formation part of the Bern model gathers the evolution of a viscously accreting gas disk, the dynamical state of the solids in the disk, the concurrent accretion of solids and gas by the protoplanets, planet–disk interactions leading to gas-driven migration and dynamical interactions between the protoplanets. Both the gas and solid disks are described by one-dimensional, axisymmetric, vertically integrated profiles. The model solves the viscous evolution equation of the gas disk^50^, with additional sink terms for the accretion by the protoplanets and external photoevaporation. The standard *α* parametrization is used to compute the viscosity, while a radiative balance is used to determine the vertical structure^51^. Solids are assumed to be in planetesimals whose dynamical state (eccentricity and inclination) is evolved due to damping by the gas disk, self-stirring and stirring by the protoplanets^52^.

Planet formation follows the core accretion paradigm with the concurrent accretion of planetesimals and gas. At time zero, 100 embryos of 0.01 *M*_⊕_ are randomly placed in the disk with a uniform probability in the logarithm of the distance between the inner edge and 40 AU. Planetesimal accretion is assumed to occur in the oligarchic regime^52^, and the planetesimal radius is set to 300 m. The model solves the one-dimensional, spherically symmetric internal structure equations^53^ to obtain either the mass or the radius of the envelope. During the early stages, gas accretion is limited by the cooling of the planet^54^ and the envelope mass is retrieved from the structure. Cooling efficiency improves as the planet grows, leading to a point where the gas accretion rate exceeds the supply from the disk, which is set according to the Bondi rate. Once this point is reached, the gas accretion is known and the internal structure equations are used to track the contraction of the envelope^55^, yielding the planet radius and luminosity.

The model prescribes gas-driven migration^34^ plus a reduction of the corotation torques due to planet eccentricity and inclination^56^. Gas-induced eccentricity and inclination damping are also included^57^. Dynamical planet–planet interactions are tracked using the mercury *N*-body code^58^.

### Improved evolution model

The formation model is coupled to an evolution model that evolves the planets individually over Gyr timescales, which is crucial to obtain realistic planetary radii. The evolution model starts at the time when the disk has dissipated. When initializing the evolution model with the planets after the formation stage, we use the luminosity the planet had at the time of disk dispersal but the mass from the time after the complete formation stage. In a test simulation, we checked the impact of this simplification, which we had to make due to the limitations of the formation model. In agreement with a recent study^59^, we found negligible differences in the final planet properties compared to starting the evolution model after 100 Myr. This is due to evolution tracks converging to the same state after Gyr timescales.

In the following, we describe in detail the changes in the evolution model compared to the previous version, which was described in full detail in ref. ^30^. The contraction and cooling of the interior structure as well as the tidal evolution remained unchanged.

The first major change concerns the treatment of water in the interior structure model. Previously, we assumed that it is always in condensed form and resides in a pure water/ice shell between the inner iron–silicate core and a possible outer gas envelope that consists of pure H/He. In the New Generation Planetary Population Synthesis (NGPPS) series^30,31^, this approximation was required, as our interior structure model was not updated for mixed H/He + H_2_O envelopes. Instead, we now allow for all phases of water—including vapour and supercritical—to exist. The orbital distance at which the valley is observed lies closer to the star than the habitable zone, meaning the temperatures at the top of the atmospheres exceed the threshold where water could condense out. Additionally, a strong runaway greenhouse occurs in vapour atmospheres at these distances^21,25^. Adding the fact that H_2_O is also miscible in H/He on a molecular level^24,25^, this implies that water does not form a separate condensed layer but is mixed into a H/He + H_2_O vapour envelope (or a pure water vapour envelope if the planet does not contain H/He). One of the latest updates to our interior structure and planet evolution model was to include such mixed compositions^60^. Specifically, we mix water described with the AQUA equation of state (EoS, see also the description below)^26^ with H/He (ref. ^61^). Our treatment of the water and H/He mix implies that the fraction of heavy elements *Z* is radially constant in the envelope. We further note that this averaged *Z* is used to calculate molecular opacities as a function of temperature but assuming Solar-like elemental abundances and equilibrium chemistry^62^.

Second, and related to the previous step, we improved the prescription for X-ray and extreme ultraviolet (XEUV)-driven atmospheric escape of the planetary envelopes. XEUV radiation drives an energy-limited (EL) mass loss rate^5^ of

1 $${\dot{M}}_{{\rm{esc}},{\rm{EL}}}={\epsilon}\frac{\pi{F}_{\rm{XEUV}}{R}_{\tau = 2/3}{R}_{\rm{base}}^{2}}{G{M}_{\rm{tot}}K({\xi} \,)},$$

where *F*_XEUV_ is the flux received in both X-ray (dominating early) and EUV wavelengths, *R*_base_ is the radius of the base of the ionization layer, *R*_*τ*=2/3_ is the radius of the optical depth *τ* = 2/3 layer, *G* is the gravitational constant, *M*_tot_ is the mass of the planet, $K(\xi \,)=1-\frac{3}{2{\xi} }-\frac{1}{2{{\xi} }^{3}}$, *ξ* = *R*_Roche_/*R*_*τ*=2/3_ the ratio of the planet’s Roche limit to its radius and *ϵ* is an escape efficiency factor. *R*_base_ is located in the planetary structure by equating the partial pressure to the pressure where an optical depth of one is reached for ultraviolet photons^63^. If this critical pressure lies exterior to the resolved envelope structure, we extrapolate using the scale height determined at 1 bar. As improvements to the NGPPS escape model^19,30^, the time evolution of *F*_XEUV_ is updated to a tabulated model based on recent observational data^64^. Furthermore, we calculate escape rates for both water and H/He separately. For water, we use equation (1) with a time (*t*)-variable *ϵ*(*F*_XEUV_(*t*)) calculated such that the mass-loss values from a one-dimensional, chemical–hydrodynamic model applied to a pure water vapour atmosphere of an Earth-mass planet in the habitable zone^28^ are reproduced. For the escape of H/He, we use extended tables based on a hydrodynamic model accounting for various heating (including XEUV) and cooling mechanisms^27^. The two rates from water and H/He escape are summed by weight: ${\dot{M}}_{{{{\rm{esc}}}},{{{\rm{total}}}}}={\dot{M}}_{{{{\rm{esc}}}},{{{{\rm{H}}}}}_{2}{{{\rm{O}}}}}Z+{\dot{M}}_{{{{\rm{esc}}}},{{{\rm{H}}}}/{{{\rm{He}}}}}(1-Z)$, where *Z* is the mass fraction of H_2_O in the envelope. When removing mass from the envelope, we assume perfect mixing and leave *Z* unchanged. This choice is motivated by the fractionation^29^ found for the pure water case^28^ in the limit of large escape rates supported by a recent work including mixed H–H_2_O (ref. ^43^). We note, however, that this simple model cannot account for effects due to the interplay of species, such as molecular cooling by H_2_O (ref. ^43,65^), which can reduce the escape rate of H/He, and that at larger planetary masses and lower metallicity, larger escape of H over H_2_O is expected^29^.

The third and final relevant variation is in the mechanism for so-called bloating, where we use an observationally derived, empirical relation^66^ in the nominal model. Bloating is the term used to describe an empirically found increase of planetary radii of mostly hot Jupiters over the expected theoretical values. To reproduce observations, an additional heat source in the deeper interior of the planetary structure is required. We model this as an additional luminosity added to the energy equation at the core–envelope boundary. In addition to the empirical model^66^, we introduce and explore variations to the bloating prescription below.

### AQUA equation of state

The AQUA equation of state is a collection of seven individual equations of state of H_2_O, which together cover a large domain in pressure and temperature useful to model planetary interiors^26^. H_2_O is a very peculiar molecule that has a large number of distinct solid phases and a complex phase diagram. Given its importance in industrial applications, H_2_O is well described by the EoS at low pressures: that is, *P* < 1 GPa. A single EoS that incorporates the many phases of H_2_O and covers the necessary large range in pressure and temperature has not yet been published. All commonly used H_2_O EoSs that cover a large range in pressure and temperature make considerable simplifications in terms of the number of treated phases and the location of the phase transitions. AQUA thus combines seven state-of-the-art EoSs into a single tabulated EoS that covers a domain from 0.1 Pa to 400 TPa in pressure and 150 K to 10^5^ K in temperature. References for each individual EoS can be found in the paper introducing AQUA^26^. Each EoS is used in a distinct region in *P–T* space, and the included phases are (1) ice-Ih; (2) ice-II, ice-III, ice-V and ice-VI; (3) ice-VII, ice-VII* and ice-X; (4) low-temperature gas, low-pressure liquid and low-pressure supercritical fluid; (5) higher-pressure supercritical fluid; (6) high-temperature gas, including ionization and dissociation of H_2_O; and (7) supercritical fluid at very high pressures, including superionic phases.

The locations of the seven regions from individual EoSs are shown in Supplementary Fig. 1. Since there are region boundaries that do not follow a physical phase transition, AQUA provides interpolated values to assure a smooth transition in all provided state parameters. The state parameters that AQUA provides for a given pressure and temperature are density *ρ*, adiabatic gradient ${\Delta }_{{{{\rm{Ad}}}}}={\left(\frac{\partial \ln T}{\partial \ln P}\right)}_{S}$, entropy *s*, internal energy *u*, bulk speed of sound *w*, mean molecular weight *μ*, ionization fraction *x*_ion_, dissociation fraction *x*_d_ and a phase identifier to identify the corresponding phase.

### KOBE

Kepler Observes Bern Exoplanets (KOBE)^23^ is a program that simulates transit surveys of exoplanets. KOBE is publicly available (github.com/exomishra/kobe). If Kepler (or TESS, PLATO and so on) was to, hypothetically, observe a synthetic population of planets then KOBE identifies those synthetic planets that would have been detected by such a survey. As a result, KOBE allows theoretical models of planet formation (such as the Bern model used here) to be compared with transit observations.

KOBE is organized in three sequential modules. The first module, KOBE-Shadow, finds transiting planets from a synthetic population of planets given their orbital elements. A planet can transit only when its orbit is aligned with respect to a hypothetical observer’s line of sight. KOBE-Transits, the next module, calculates transit parameters. It applies the detection biases coming from physical limitations; large planets in tight orbits around a quiet star are strongly favoured. For comparison to Kepler, transiting planets that transit at least three times and have a signal-to-noise ratio ≥7.1 are potentially detectable. KOBE-Vetter, the final module, applies the completeness and reliability of the Kepler pipeline by emulating Kepler’s Robovetter^67^. Transiting planets that are identified as planetary candidates by KOBE-Vetter make up the KOBE-biased catalogue that we use for comparison to the Kepler-based California Kepler Survey results^2^. The planets in the KOBE catalogue are comparable to the exoplanet population discovered by Kepler.

### Impact of the evaporation model

Here, we investigate the effect of several key model assumptions on the final planet properties. We use the same planetary population after the formation stage but change the used description for the effects of photoevaporation and bloating of the planetary envelopes.

As a first test, we see that removing the effect of photoevaporation results in a distribution of radii without a valley. Photoevaporation is a necessary process to populate the envelope-stripped, super-Earth peak (Fig. 2), with planets located close to or within the observed radius valley otherwise. Furthermore, without photoevaporation, planets in the super-Earth regime would commonly contain large amounts of water, which is not realistic at these planetary masses and distances to the star^37^.

To model photoevaporation, we nominally used the tabulated evaporation rates based on recent detailed radiation–hydrodynamic models for H/He-dominated^27^ and water-dominated^28^ envelopes. To contrast them to a more simple model, we show in Supplementary Fig. 2b the resulting distribution of planetary radii using energy- and radiation-recombination-limited escape rates^5,19^ and further, in Supplementary Fig. 2c, a variant thereof that accounts for an increase in mean molecular weight as a function of the envelope metallicity *Z* when calculating the base layer of the evaporative flow and a scaling of the efficiency factor *ϵ* with *Z* in equation (1) taken from ref. ^37^. While the former gives results similar to the nominal evaporation model, the *Z*-dependent version results in a less pronounced super-Earth peak as fewer planets lost their primordial envelope.

For reference, we also show the resulting planetary masses in Supplementary Fig. 3 for the same model variations. A shifted overall peak at higher masses is found in mass space when removing the effect of photoevaporation (Supplementary Fig. 3f) but no significant differences result based on the different evaporation model.

### Impact of bloating

Since bloating of the envelope leads to an increase of the planetary radius and therefore a larger surface area irradiated by the star, positive feedback between bloating and photoevaporation of the planets is expected. This warrants exploring an alternative model based on the physical potential temperature advection mechanism^68^ instead of the nominal, empirically derived bloating model used in the main analysis^66^. We further investigate the effects on the population of synthetic planets in the absence of bloating. The latter exploration is interesting if the so-far-unknown bloating mechanism is not acting on lower planetary masses in the super-Earth and sub-Neptune regimes and would instead apply only to giant planets.

### Bloating model using a fit to the potential temperature advection model

To include potential temperature advection as a bloating mechanism^68^, we add a luminosity term at the core-mantle boundary in the interior structure calculation. We start with a literature fit of the temperature at the layer where a pressure of 100 bar is reached. The corresponding temperature is a function of the stellar irradiation flux *F* (ref. ^68^). We further subtract 200 K, motivated by the results from three-dimensional simulations^69^. We then calculate the entropy and, using our interior structure models, the required bloating luminosity *L*_bloat_ to match this entropy level.

To take into account the mass fraction of heavy molecules (that is, water) in the envelope *Z*, we use a *Z-*dependent fit of the form

2 $${L}_{{{{\rm{bloat}}}}}(F,Z\,)=a(Z\,)+b(Z\,){\log }_{10}F+c(Z\,){\log }_{10}^{2}F,$$

where *F* is the irradiation flux. To obtain results for continuous values of *Z*, we interpolate linearly between the fits, and we further found a linear dependency of the bloating luminosity on planetary mass to obtain the desired entropy. The resulting parameters for equation (2) are listed in Supplementary Table 1.

### Inconclusive evidence between bloating models

In Supplementary Fig. 2, we show resulting distributions of planetary radii for the nominal, empirical bloating model^66^ (Supplementary Fig. 2a), for the model with radius inflation caused by the potential temperature advection mechanism^68^ (Supplementary Fig. 2d) and without any bloating mechanism (Supplementary Fig. 2e). If included, we assume that the bloating mechanism is acting on all planetary masses, and we see a small impact of the bloating mechanism on the sub-Neptune population (blue histogram). It is expected that bloating by ohmic dissipation is efficient in sub-Neptunes^70^. Without a bloating mechanism, the valley would be more populated by smaller water-rich planets, therefore making it less deep and visible. However, the effect is too small to be constrained by the current observational data. Both compared bloating models give very similar results. Overall, we find that the presence of a radius valley is robust against uncertain extrapolations of the radius-inflation mechanism to lower planetary masses and different compositions.

### Supplementary information

Supplementary Information Supplementary Figs. 1–3 and Table 1.

## Data Availability

Raw data used in this study are accessible at https://dace.unige.ch/ under identifier NG76. Derived data supporting the findings of this study are publicly available at 10.5281/zenodo.7646318. Supplementary data are available upon reasonable request.

## Code availability

The code for the statistical analysis and to generate all figures of the manuscript is available at 10.5281/zenodo.7646318. The modified planetary evolution code will be published in open access in the future and is available on reasonable request.
