# Supplementary material for: A radius valley between migrated steam worlds and evaporated rocky cores
Source: Nat Astron. 2024 Feb 9;8(4):463–71. doi: 10.1038/s41550-023-02183-7 (PMC11035145; doi:10.1038/s41550-023-02183-7)
Supplement: Supplementary file 1 — Supplementary Figs. 1–3 and Table 1. [file 41550_2023_2183_MOESM1_ESM.pdf]

nature astronomy

Article

https://doi.org/10.1038/s41550-023-02183-7

A radius valley between migrated steam worlds and evaporated rocky cores

In the format provided by the authors and unedited

Contents

1 Supplementary figures **1**
1.1 Equation of State 1
1.2 Additional simulation results 2

2 Supplementary tables **4**
2.1 Model parameters 4

1 Supplementary figures

1.1 Equation of state

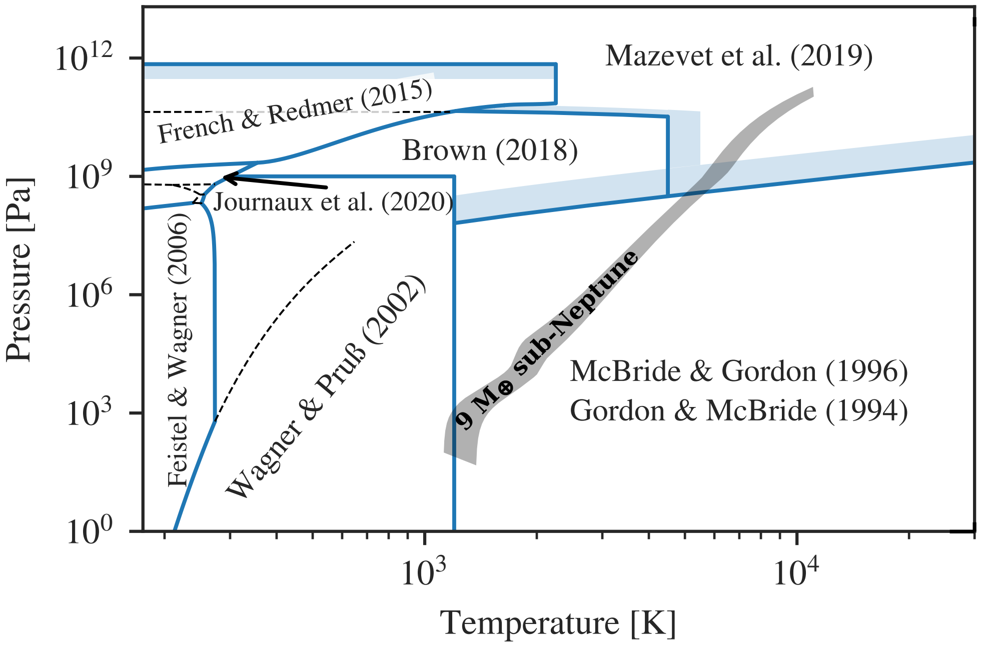

Figure 1: Phase diagram of H_2O split into the seven regions, separated by the solid blue lines. In each region a different EoS is used. Most region boundaries follow phase transition curves of H_2O. The dashed lines are phase transitions that are not region boundaries, meaning the same EoS is used along the phase transition. The blue shaded areas show where neighboring regions have to be interpolated to achieve smooth transitions in the state parameters. The time evolution of a sub-Neptune water envelope at 3 days orbital period with an initial mass of 2.7 $M_\oplus$ on top of a rocky 6.3 $M_\oplus$ core is shown in gray. Its inner layers are supercritical at high pressure and the upper layers mostly in the high temperature gas regime.

1.2 Additional simulation results

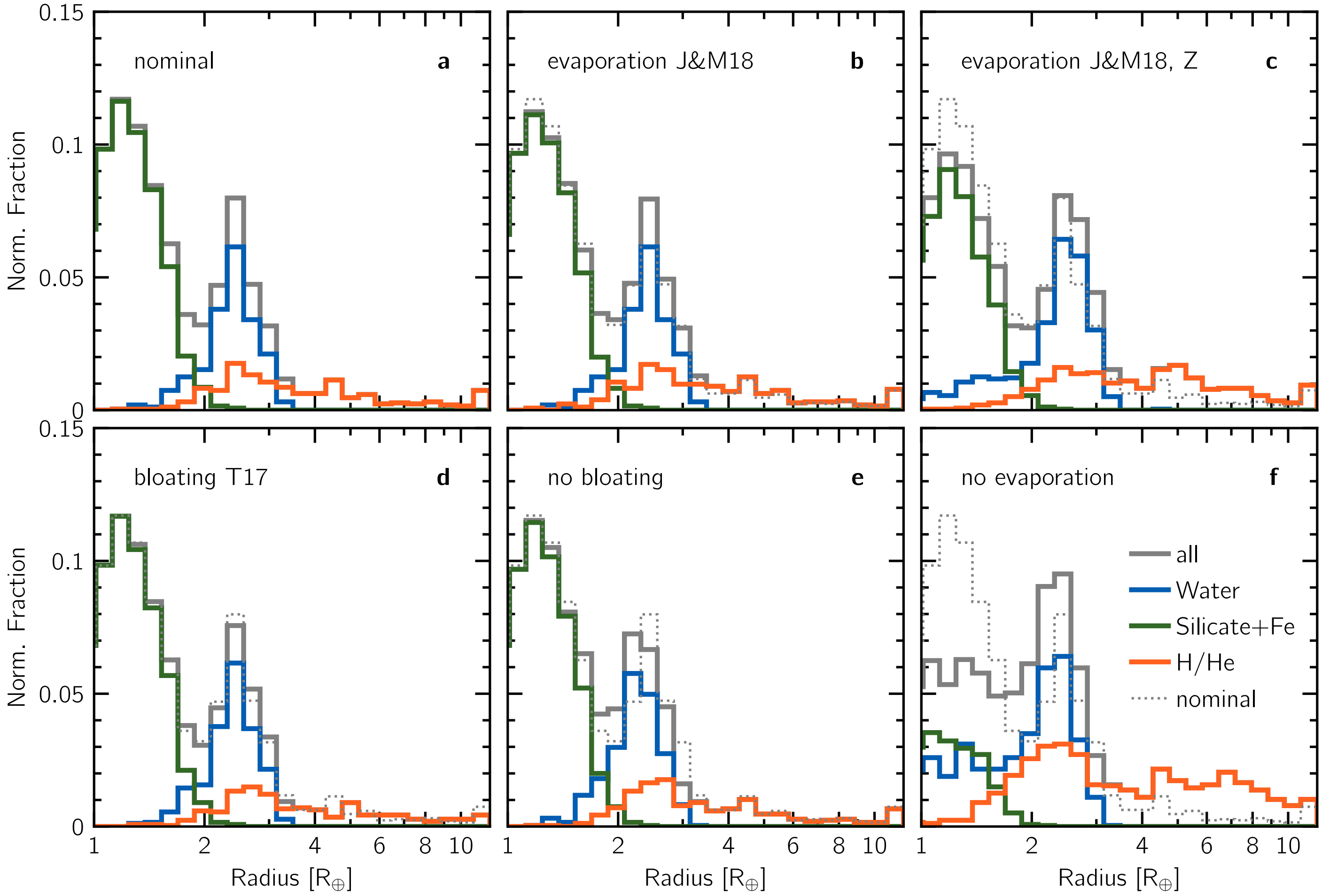

Figure 2: Histogram of planetary radii for different assumptions in the evolution model. All planets with orbital periods less than 100 d are included. The colored histogram are the same as in Fig. 2.

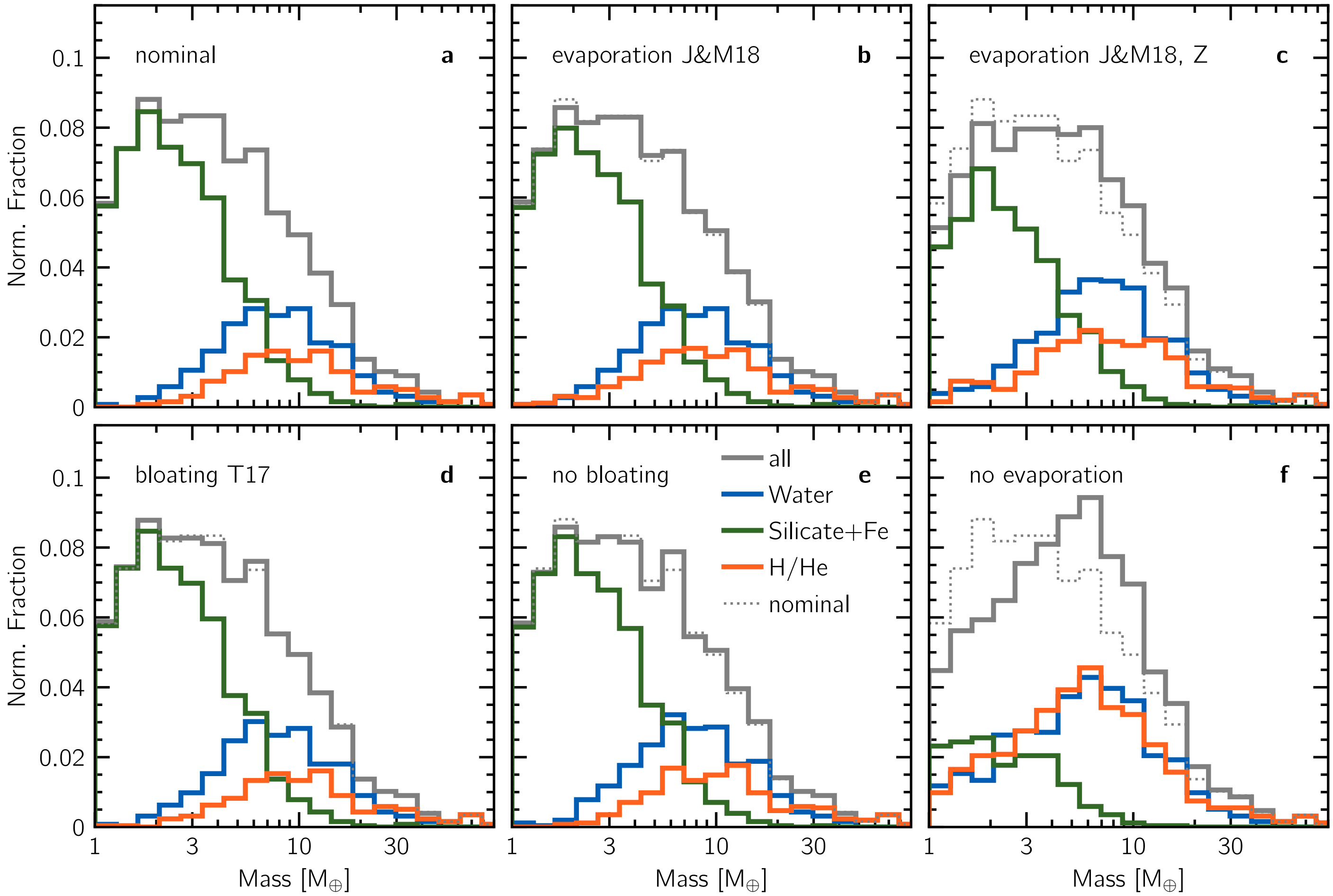

Figure 3: Histogram of planetary masses for different assumptions in the evolution model. The selection of planets and colors are identical to Fig. 2.

2 Supplementary tables

2.1 Model parameters

Table 1: Fit parameters for Eq. 2 for a planet like HD209458b (241.9 $M_{\oplus}$)

Envelope enrichment	*a*	*b*	*c*
Z = 0	-0.555	-0.171	0.066
Z = 0.2	-9.581	1.881	-0.061
Z = 0.4	-23.940	5.311	-0.267
Z = 0.6	-41.709	9.610	-0.525
Z = 0.8	-61.618	14.462	-0.818
Z = 1.0	-82.456	19.606	-1.132
